# *In-vitro* analysis of Quantum Molecular Resonance effects on human mesenchymal stromal cells

**DOI:** 10.1371/journal.pone.0190082

**Published:** 2018-01-02

**Authors:** Sabrina Sella, Valentina Adami, Eliana Amati, Martina Bernardi, Katia Chieregato, Pamela Gatto, Martina Menarin, Alessandro Pozzato, Gianantonio Pozzato, Giuseppe Astori

**Affiliations:** 1 Advanced Cellular Therapy Laboratory, Hematology Unit, Vicenza Hospital, Vicenza, Italy; 2 High Throughput Screening Core Facility, Center for Integrative Biology, University of Trento, Trento, Italy; 3 Hematology Project Foundation, Vicenza, Italy; 4 Telea Electronic Engineering srl, Sandrigo, Italy; Universita degli Studi di Udine, ITALY

## Abstract

Electromagnetic fields play an essential role in cellular functions interfering with cellular pathways and tissue physiology. In this context, Quantum Molecular Resonance (QMR) produces waves with a specific form at high-frequencies (4–64 MHz) and low intensity through electric fields. We evaluated the effects of QMR stimulation on bone marrow derived mesenchymal stromal cells (MSC). MSC were treated with QMR for 10 minutes for 4 consecutive days for 2 weeks at different nominal powers. Cell morphology, phenotype, multilineage differentiation, viability and proliferation were investigated. QMR effects were further investigated by cDNA microarray validated by real-time PCR. After 1 and 2 weeks of QMR treatment morphology, phenotype and multilineage differentiation were maintained and no alteration of cellular viability and proliferation were observed between treated MSC samples and controls. cDNA microarray analysis evidenced more transcriptional changes on cells treated at 40 nominal power than 80 ones. The main enrichment lists belonged to development processes, regulation of phosphorylation, regulation of cellular pathways including metabolism, kinase activity and cellular organization. Real-time PCR confirmed significant increased expression of MMP1, PLAT and ARHGAP22 genes while A2M gene showed decreased expression in treated cells compared to controls. Interestingly, differentially regulated MMP1, PLAT and A2M genes are involved in the extracellular matrix (ECM) remodelling through the fibrinolytic system that is also implicated in embryogenesis, wound healing and angiogenesis. In our model QMR-treated MSC maintained unaltered cell phenotype, viability, proliferation and the ability to differentiate into bone, cartilage and adipose tissue. Microarray analysis may suggest an involvement of QMR treatment in angiogenesis and in tissue regeneration probably through ECM remodelling.

## Introduction

Cells interact with the surrounding environment through receptors and ion channels which transmit chemical, mechanical and electrical signals. In this context, electromagnetic fields (EMF) interfere with cellular pathways and tissue physiology [1]. Cell-EMF interaction can occur through charged molecules and proteins in the cell membrane that alters the flow of ions or rearranges the distribution of the membrane receptors, or via direct field penetration inside the cell [2].

There is evidence that the manipulation of the electromagnetic environment on biological systems favours wound healing process, reduction of inflammatory state, angiogenesis and extracellular matrix (ECM) synthesis [3]. In fact, EMF regulate a variety of cell functions including promotion and inhibition of cellular proliferation [4, 5], cellular viability [6, 7], differentiation [8–10], cellular migration and motility [11–13], inflammatory response [14, 15] and gene expression profiles [16, 17]. As a consequence, therapeutic application of EMF has undergone a raising interest in medicine. By contrast, the mechanisms of action of EMF in biological tissues are only partially known [18].

Quantum Molecular Resonance (QMR) stimulation is a technology already applied for surgical and medical purposes. QMR creates quanta of energy able to break the molecular bonds without increasing the kinetic energy of the hit molecules, thus without raising in the temperature and limiting the damage to the surrounding tissue. QMR technology exploits no-ionizing high-frequency waves in the range between 4 and 64 MHz at low intensity delivered through alternating electric fields. The effect of QMR stimulation relies on the induction of more frequencies at the same time, where the fundamental wave is at 4 MHz and the subsequent ones increase in harmonic content until 64 MHz with related decreasing amplitudes.

QMR finds clinical application in bipolar coagulators or electrosurgery devices [19, 20]. For this kind of applications, the molecular resonance generator works on the combination of four frequencies in the range of 4–16 MHz. The first experimental study testing QMR effects described in a rat model of thoracotomy a less severe tissue damage than standard electrocautery [21].

Since now, few data are available on the mechanism of interaction between QMR and cells. Dal Maschio and colleagues [22] provided the description of the behavior of muscle fibers exposed to QMR, where the changes of membrane potential and the variations of free calcium concentration strictly followed the time course of electrical field application and removal. Moreover, the effectiveness of molecular quantum resonance in reducing edema after total knee arthroplasty in a clinical trial has been reported [23].

Our work aimed at understanding how QMR acts on human bone marrow-derived mesenchymal stromal cells (MSC).

The use of MSC for tissue healing and in regenerative medicine was extended in the last decade [24], but current research on MSC aims not only to the development of clinical protocols of cellular therapy or regenerative medicine but also to provide experimental models that can inform about molecular mechanisms such as inflammation, angiogenesis and apoptosis [25].

Three main criteria were proposed by the International Society for Cellular Therapy (ISCT) for MSC definition [26]: adherence to plastic under standard culture conditions; expression of CD105, CD73, CD90 and lack of expression of HLA-DR, together with the lack of hematopoietic and endothelial surface markers CD14, CD45, CD34, CD11b and CD31 [27]; *in vitro* differentiation potential into osteocytes, chondrocytes and adipocytes under appropriate culture conditions [28]. Despite of attempts for establishing generally acceptable minimal criteria for defining human MSC by immunophenotyping, the functional capability to differentiate along the classical tri-lineage mesodermal pathways remains one fundamental characteristic of this cell type.

MSC represent an ideal model to study the effects of high frequency EMF and electric current. MSC exhibit remarkable plasticity given their ability to transdifferentiate or undergo rapid alteration in phenotype, thereby giving rise to cells possessing the characteristics of different lineages. Moreover, there are evidences that endogenous bone marrow-derived MSC could be recruited and mobilized to sites of injury [29]. As a consequence MSC can be used in various clinical conditions in which tissue repair is needed, or in which these cells are believed to act through their anti-inflammatory and immunomodulatory activities.

In the present study, we used a broad evaluation approach to study the effects of QMR on human MSC at different levels of investigation. Cell cultures were exposed to distinct QMR settings and times of treatment. We assessed the maintenance of MSC identity and then therefore performed viability and cellular proliferation assays to assess additional information about. Finally, we investigated the transcriptional profile of MSC after QMR stimulation.

## Materials and methods

### MSC isolation and *ex-vivo* expansion

MSC were isolated from cells obtained through the washouts of discarded bone marrow collection bags and filters of healthy donors, 2 male and 4 female (median age: 34.5 years. Range 23–47). After two washing steps with 200 ml saline solution and centrifugation at 2.000 rpm for 10 min, the collected nucleated cells were seeded in toto at the density of 1x10^5^ cells/cm^2^ in low-glucose Dulbecco’s modified Eagle’s medium (DMEM) with GlutaMAX^TM^ and pyruvate (Gibco, Thermo Fisher Scientific, Waltham, MA, USA) supplemented with 10% fetal bovine serum (FBS, Qualified Australian, Gibco, Thermo Fisher Scientific) and 1% penicillin/streptomycin (P/S, Sigma-Aldrich, St Louis, MO, USA). Cultures were incubated at 37°C in a humidified atmosphere with 5% CO_2_. Non-adherent cells were removed after 72 hours and fresh medium was added, then the culture medium was changed every 3–4 days. At 80% confluence, MSC were washed with Dulbecco’s phosphate-buffered saline (D-PBS, Sigma-Aldrich), harvested using 10X TrypLE Select (Gibco, Thermo Fisher Scientific) and sub-cultured at a density of 1.500 cells/cm^2^. The cultures were observed with an inverted light microscope Axiovert 40 CFL (Carl Zeiss, Oberkochen, Germany) and the images were acquired by using an AxioCam Mrm camera system (Carl Zeiss).

### Cellular model and QMR stimulation protocol

MSC cultures were exposed to QMR using an experimental QMR generator supplied by Telea (Telea Electronic Engineering, Sandrigo, VI, Italy). The QMR generator setup was the following: alimentation: 230 V ~ 50/60 Hz; maximum power in input: 250 VA; power in output: 45 W/400 Ω. The prototype enhanced alternating electric currents characterized by high-frequency waves and low intensity. The fundamental wave was at 4 MHz and the subsequent ones increased in harmonic content until 64 MHz with related decreasing amplitudes. The stimulations were delivered through the raise of effective powers in output (4–45 W) corresponded to increase in value to the nominal powers employed as QMR settings.

**Fig 1:**
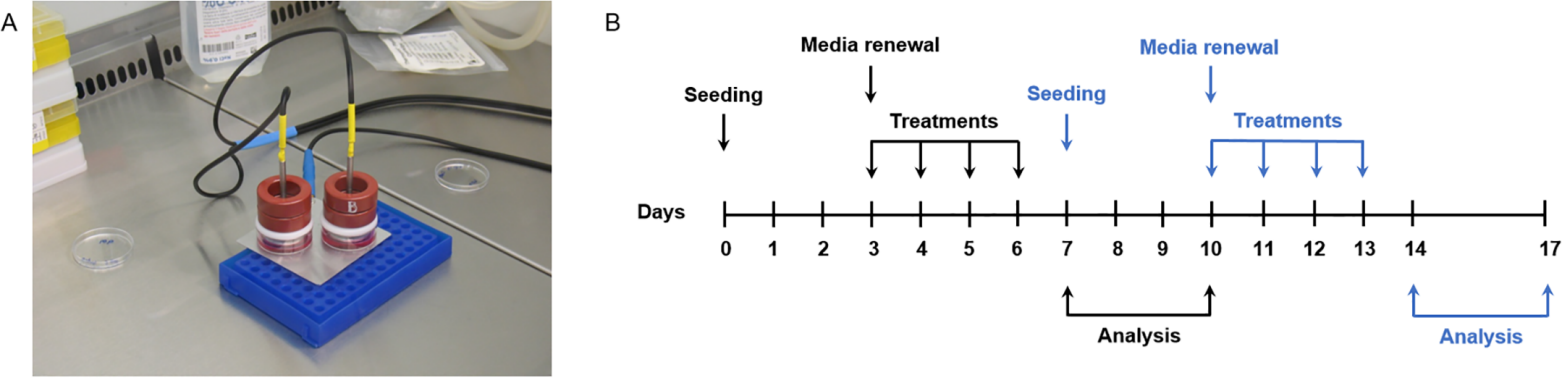
QMR stimulation protocol. A) Image of the exposure system. B) Scheme of QMR treatment. Cells were seeded on day 0, harvested and reseeded on day 7. The first cycle of treatment started after media renewal on day 3 (black arrows) and the second one on day 10 (blue arrows). Cultures were stimulated 10 minutes/day for 4 consecutive days at 40 or 80 nominal powers. Sham-exposed controls were kept in parallel.

The cellular model and QMR delivery system were composed of a pair of custom made spheroidal electrodes (anodes) of 35 mm-diameter and by an electrode (cathode) constituted of a metallic plate. The electrodes were placed inside two Petri dishes and supported by a polyvinyl chloride component to allow the direct contact of the electrode with the surface of the culture medium. The cathode was positioned below the Petri dishes (Fig 1A).

The experimental setup was planned in order to reproduce in-vitro the therapeutic conditions in terms of timing and powers. Based on medical reports and on long company experience, the most effective settings with positive follow-up were selected for MSC cultures experimentation.

MSC at passages 4–6 were seeded in 35 mm-diameter Petri dishes in duplicate per condition (Greiner Bio-One, Kremsmünster, Austria) and after 72 hours from initial seeding, complete medium was changed. Cells were subjected to 10 minutes/day of QMR stimulation for 4 consecutive days with rest period of 24 hours between them. The same MSC cultures exposed to the first QMR cycle of treatment were reseeded for the second one and treated under identical conditions (Fig 1B). Two different QMR settings corresponding to 40 and 80 nominal powers were applied. Controls were kept in parallel as sham-exposed controls with electrodes presence in cell media and without QMR exposition.

### MSC phenotype characterization

MSC phenotype was characterized by flow cytometry before and after QMR stimulation. Briefly, 1x10^5^ cells were incubated with the following monoclonal antibodies: CD90-FITC, CD105-PE, CD45-ECD, HLA-DR-APC (all purchased from Beckman Coulter, Brea, CA, USA) and CD73-PC7 (Becton Dickinson, Franklin Lakes, NJ, USA) for 15 minutes at room temperature protected from light. At least 20.000 events were acquired on a CYTOMICS FC500 flow-cytometer and data were analysed by Kaluza software (both Beckman Coulter). The expression of each marker was assessed as percentage (%) of positive cells and as relative median fluorescence intensity (rMFI), this latter defined as the ratio between the median fluorescence intensity of the marker and its specific negative control.

### Multilineage differentiation

After two cycles of consecutive stimulations MSC differentiation potential was tested. Samples were harvested and reseeded in 24-well plates (Falcon, Corning Life Sciences, NY, USA) in the presence of circular 13 mm-diameter and 0.2 mm-thickness coverslips (Nunc, Thermo Fisher Scientific) at the density of 4.000 cells/cm^2^. Differentiation was induced at semi-confluence with specific differentiation media for 21 days with StemPro Adipogenesis, Osteogenesis and Chondrogenesis kit, (Gibco, Thermo Fisher Scientific). Fresh medium was added every 3 days and the respective controls were maintained in parallel with standard expansion medium.

To detect the formation of lipid droplets, cells were fixed in 10% formalin for 5 minutes and stained with Oil Red O (Diapath, Martinengo, BG, Italy) according to manufacturer’s instructions.

The presence of calcium deposit as an expression of osteogenic induction was analysed with Alizarin red staining. The samples were washed with D-PBS and fixed in ice-cold 70% ethanol at 4°C for 1 hour. Then, they were incubated for 15 minutes with 0.02 g/ml of Alizarin red solution (Sigma-Aldrich) at room temperature. Finally, several washes were performed with deionized water.

To verify chondrogenic differentiation, cells were fixed in 10% formalin for 5 minutes and stained with 1 g/l in 0.1 M HCl of Alcian blue for 2 hours at room temperature. At the end of the staining specific for acidic polysaccharides, the coverslips were rinsed extensively with deionized water.

After each staining, the coverslips were mounted on microscope slides using Kaiser’s glycerol gelatine pre-warmed at 37°C. The acquisition of images was obtained by AxioCam Erc 5s camera system (Carl Zeiss).

### Assessment of cellular viability

Cellular viability was determined by flow cytometry using LIVE/DEAD Fixable Far Red Dead Cell Stain kit (Invitrogen™, Thermo Fisher Scientific) according to manufacturer’s instructions by samples acquisition on a CYTOMICS FC500 flow-cytometer. Data were analysed as % of dim or bright positive cells by Kaluza software (Beckman Coulter).

### Quantification of cellular proliferation

Cellular proliferation was determined by WST-1 assay (Sigma Aldrich). At the end of two consecutive cycles of QMR treatment, cells were harvested and seeded in 96 well-plates (Falcon, Corning Life Sciences) at the density of 2.000 cells/well. After 72 hours WST-1 was added and incubated for 3 hours at 37°C. Finally, the plates were read at 450 nm with a spectrophotometer (SpectraCount, Packard Instrument Company Inc, Meriden, CT, USA). Data were expressed as percentage (%) of proliferation on the control.

### cDNA microarray analysis

The RNA derived from five different MSC samples exposed to one cycle of QMR stimulation and their corresponding MSC controls were extracted using RNeasy plus mini kit (Qiagen, Hilden, Germany) according to the manufacturer’s instructions. The total RNA quantification was obtained with NanoDrop UV-VIS Spectrophotometer (Thermo Fisher Scientific). The quality of RNA was determined using Agilent 2100 Bioanalyzer system with Eukaryote Total RNA Nano kit (Agilent Technologies, Santa Clara, CA, USA). The samples were processed according to protocol “Agilent One-Color Microarray-based Gene Expression Analysis (Low Input Quick Amp Labeling)” with Human GE 4x44K V2 Microarray Kit (Agilent Technologies).

Microarray slides were detected with Agilent scanner through ScanControl software. Row data from microarray images were extracted by Agilent Feature Extraction software. Afterward data were subjected to a pre-processing step using open-source program Bioconductor that employs the Limma package with R language [30].

### Quantitative real-time PCR

MSC cultures were exposed or not to QMR at 40 nominal power for one cycle and the total RNA was extracted using RNeasy Plus Mini Kit (Qiagen) following the manufacturer’s instructions. Quality and quantity were determined using Nanodrop UV-VIS Spectrophotometer (Thermo Fisher Scientific). cDNA was synthesized starting from 800 ng of total RNA, using the iScript cDNA synthesis kit (Bio-Rad, Hercules, CA, USA) according to the manufacturer’s instructions.

The obtained cDNA was diluted 1:10 and the quantitative real-time PCR experiments were performed using SsoFast EvaGreen Supermix with Low Rox (Bio-RAD) on ABI 7500 Real-Time PCR System (Applied Biosystem, Thermo Fisher Scientific). Primers used for the amplification were validated and purchased from Bio-RAD. The protocol consisted of 30 seconds at 95°C, 40 cycles of 5 seconds at 95°C and an elongation step of 32 seconds at 60°C, followed by a final melting step to evaluate the quality of the product. Each gene was tested in three replicates and six independent experiments were performed. Data acquisition was obtained by SDS v1.2 software (Applied Biosystem, Thermo Fisher Scientific) and the relative expression was determined using 2^-ΔΔCt^ method [31] with TBP and YWHAZ as reference genes.

### Statistical analysis

To analyse the differences between the experimental settings and the sham-exposed controls after both QMR cycles, data were analysed by one-way ANOVA followed by Dunnett's multiple comparison post-hoc test.

Quantitative real-time PCR data were analysed by paired t-test comparing the ΔCt values. Statistical analysis was performed using GraphPad Prism version 5.01 (GraphPad Software Inc, La Jolla, CA, USA). Differences between samples were considered statistically significant at p<0.05.

For the cDNA microarray analysis, differentially expressed genes (DEG) between treated (40 or 80 nominal power) cells and control cells were elaborated using Limma package with Bayes’ empirical method, taking into account the provenience of batches (paired test). Differences between conditions (treated cells versus control cells) were considered significant after Benjamini & Hochberg correction at p<0.05. To analyse the best enrichment of gene lists, the ToppGene Suite and Ingenuity Pathway Analysis (IPA) computational tools were applied. It was considered a q-value<0.01 with Benjamini & Hochberg’s false discovery rate (FDR) correction for ToppGene Suite and a p-value<0.01 with Bonferroni-Hochberg correction for IPA analysis.

## Results

### Evaluation of MSC identity after QMR stimulation

**Fig 2:**
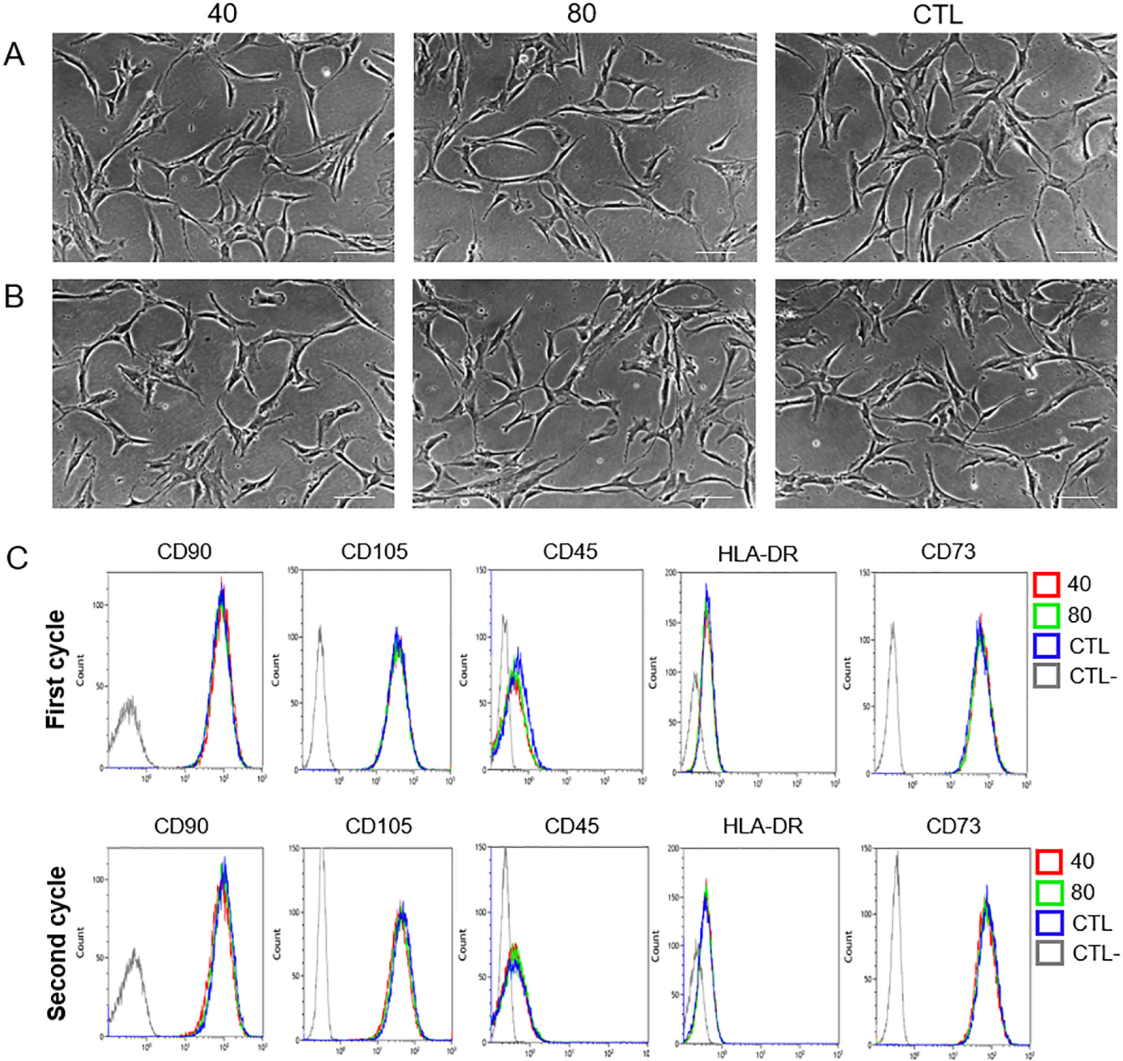
MSC morphology and flow cytometry analysis after QMR stimulation. A) The images were obtained after 10 minutes of QMR stimulation at Day 5 (first cycle of treatment) and at B) Day 12 (second cycle of treatment). Scale bar = 100 μm. Total magnification = 100X. One representative experiment was shown. C) Five colour combination of monoclonal antibodies was used to verify MSC identity according to the above listed surface markers of a representative sample. Grey line = unstained control (CTL-). Blue line = sham-exposed control (CTL). Green line = QMR setting 80. Red line = QMR setting 40.

**Fig 3:**
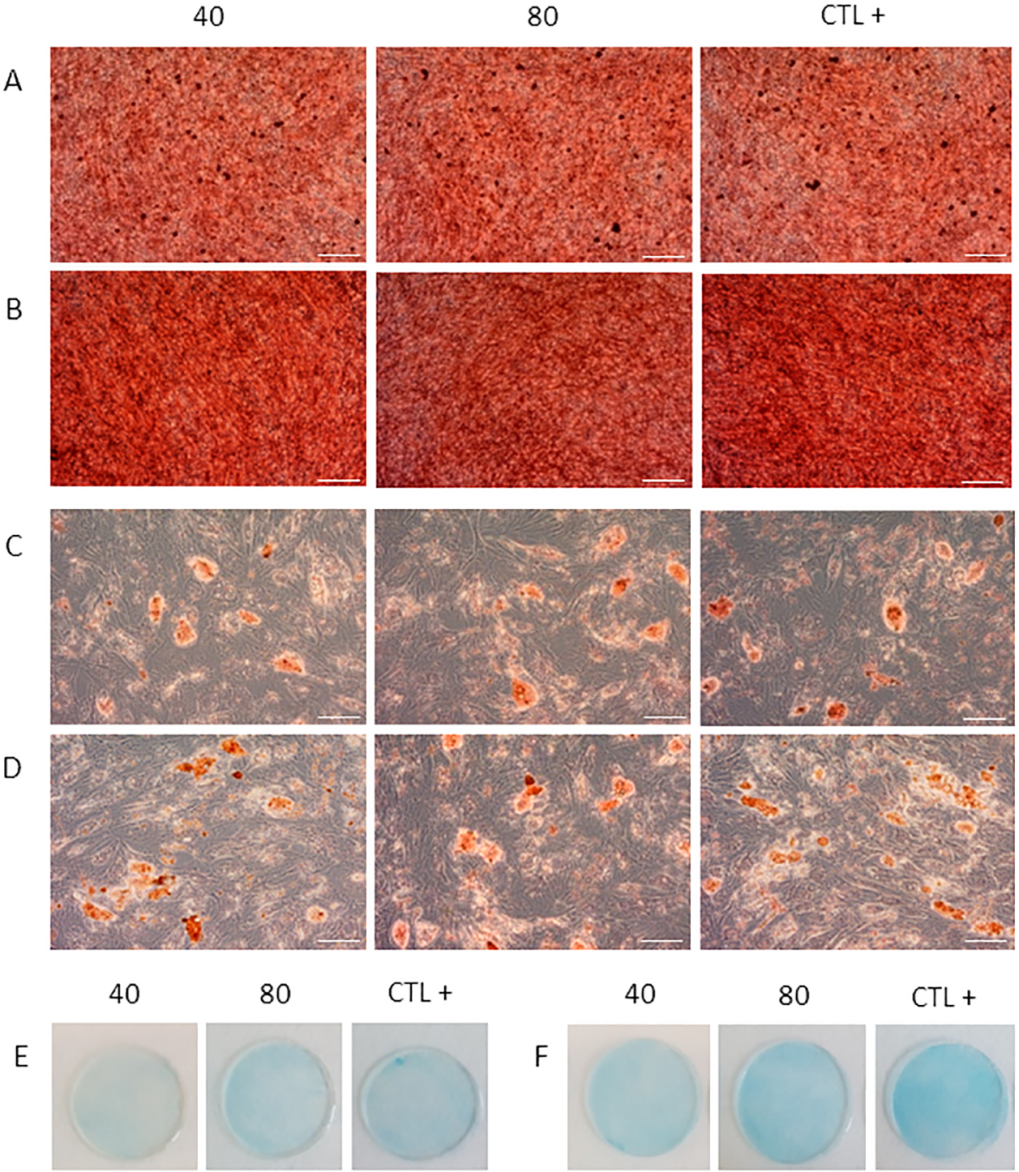
Adipogenic, osteogenic and chondrogenic differentiation after QMR cycles of stimulation. Panels display one representative experiment showing the final outcome in MSC multilineage differentiation after 21 days of induction. QMR-treated (at 40 and 80 nominal powers) and untreated samples (CTL+) were induced to differentiation. Osteogenic differentiation after one cycle (A) and two cycles (B) of QMR stimulation was assessed using Alizarin Red. Adipogenic (C, D) and chondrogenic (E, F) differentiation were detected using Oil Red O and Alcian Blue stainings, respectively. Scale bar = 100 μm. Total magnification = 100x.

In order to evaluate modification in MSC identity after the first and second cycle of treatment with QMR, we analysed morphology, expression of surface markers and multi-differentiation potential. MSC morphology was observed daily before and after QMR treatments at the different settings. The cells conserved their canonical fibroblast-like spindle-shaped aspect during all the time of the experiments (Fig 2A and 2B). Typical phenotypic MSC expression of CD90, CD105 and CD73 was constantly >95%, while that of CD45 and HLA-DR constantly lower than 2% (Fig 2C). Moreover each marker showed similar rMFI of expression without statistical significance between treated and untreated samples at the different settings (S2 Fig). To investigate if cell cultures lost their *in vitro* mesenchymal differentiation potential after two cycles of QMR stimulation, we induced cells to differentiate down into osteogenic, adipogenic and chondrogenic lineages by using defined media components and conditions (Fig 3). QMR-treated and sham-exposed MSC samples were able to multi-differentiate after 21 days of induction, being positive to Alizarin Red, Oil Red O and Alcian blue specific staining for osteogenesis (Fig 3A and 3B), adipogenesis (Fig 3C and 3D) and chondrogenesis (Fig 3E and 3F), respectively. No qualitative differences were observed between different QMR treatments and controls at this level. Similar results were detected between one (Fig 3A, 3C and 3E) and two cycles (Fig 3B, 3D and 3F) of stimulation.

### MSC viability and proliferation after QMR stimulation

**Fig 4:**
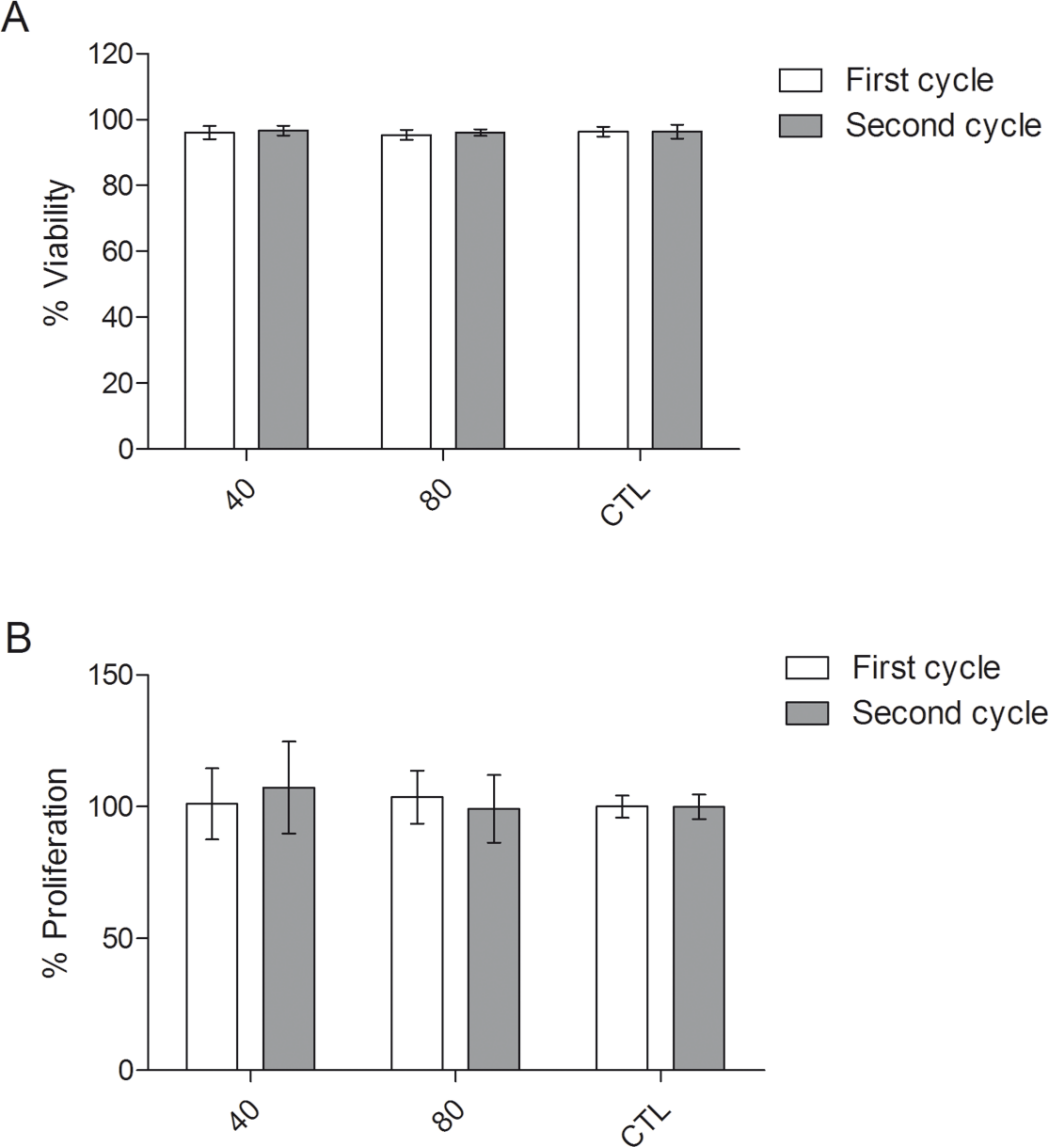
Cellular viability and proliferation after QMR treatment. A) Histograms represent the % of cellular viability after two cycles of QMR treatment at the different settings compared to the sham-exposed controls determined by flow cytometry. Data were shown as mean ± SD of three independent experiments; B) Percentages of cellular proliferation on the controls were obtained by WST-1 assay after 72 hours. Data were represented as mean ± SD of n = 6 independent experiments. No statistical differences were found between conditions.

Cellular viability was quantified by flow cytometry at the end of each cycle (Fig 4A). Viability was not affected by QMR; indeed, more than 95% of cells were alive similarly to the controls, with low variability between the different MSC batches and settings. These results confirmed the morphological observations. MSC proliferation was not affected by QMR showing no significant differences between controls and QMR-treated samples at the different settings and times (Fig 4B).

### Microarray gene expression analysis after QMR treatment

Based on previous results, we studied the effect of QMR on MSC at transcriptional level by performing cDNA microarray experiments after one cycle of treatment (Day 7). cDNA microarray data pre-processing step reduced the initial number of transcripts from 28,000 to about 12,600. After that, samples were grouped by the similarity of gene expression profiles (doi: 10.6084/m9.figshare.5702137). Not surprisingly, results of clustering showed that samples grouped mainly according to the donor’s provenience and not by QMR treatments, as a result of inherent biological variability between analysed MSC batches.

**Fig 5:**
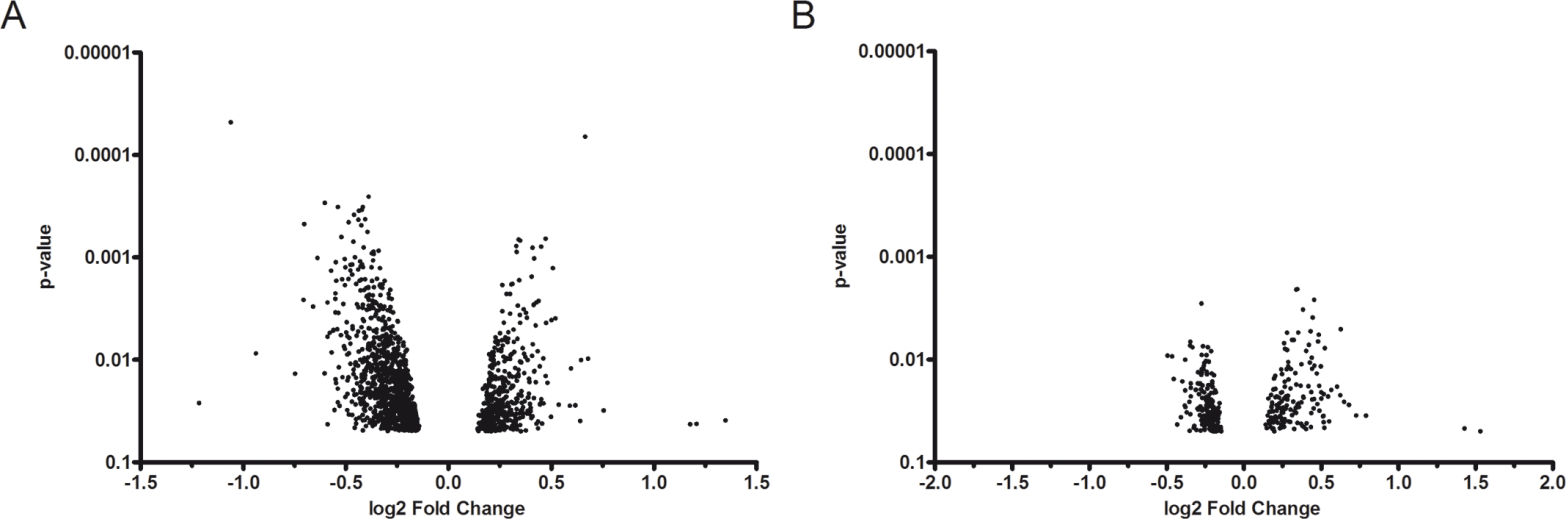
Dot plots of differentially expressed genes associated with QMR treatment at 40 and 80 nominal powers. Images illustrated the distribution of (A) 40 and (B) 80 up- and down-regulated genes (Benjamini & Hochberg correction at p<0.05). The results were assessed by comparing QMR-treated MSC (after one cycle of QMR at 40 and 80 nominal powers) with untreated control cultures. The y-axis was in log10 scale.

DEG analysis was applied to identify the differences between QMR-treated and sham-exposed MSC samples. Three out of the 16 samples (15 samples + 1 technical replicate) did not meet quality control criteria and therefore were discarded from the subsequent analysis. More transcriptional changes were identified for 40 nominal power than 80 ones. According to a cut-off corrected p-value<0.05, 411 up-regulated and 987 down-regulated genes were found when using 40 as nominal power (Fig 5A). At 80 nominal power, 163 genes were found up-regulated while 199 down-regulated (Fig 5B). In both cases, most of the DEGs showed a very low fold change.

**Fig 6:**
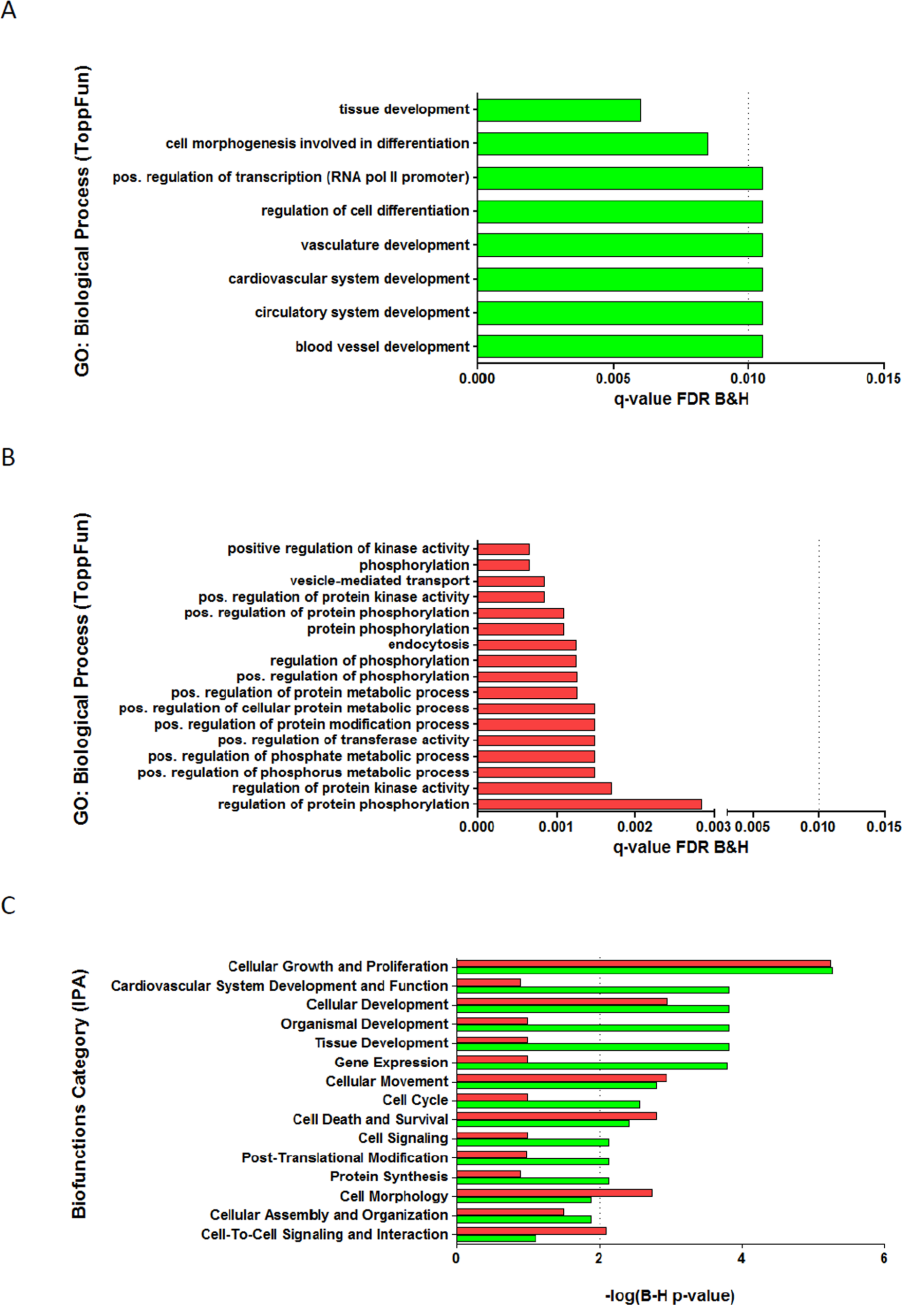
Best enrichment gene lists. Analysis of functional gene enrichment using ToppFun tool (application of ToppGene Suite) for A) up-regulated and B) down-regulated DEG between 40 QMR setting and control with significant enrichment (dotted line) for FDR B&H q-value <0.01); C) Comparative analysis of up-regulated (green bar) and down-regulated (red bar) functional gene enrichments using IPA software with significant enrichment (dotted line) for -log2 (B-H p-value) >2.

To investigate the biological processes and biofunctions in response to QMR stimulation, gene enrichment analysis was performed using ToppGene Suite and IPA tools (Fig 6). The main biological processes up-regulated by 40 nominal power were related to cellular and tissue development, cellular differentiation and vascular system development (Fig 6A and 6C). Positive regulation of protein phosphorylation, vesicle mediated-transport, positive regulation of metabolic processes (Fig 6A), cellular morphology and cell-to-cell interaction biofunctions (Fig 6C) were found down-regulated by the QMR stimulation. Cellular proliferation and movement processes are equally significantly enriched in both gene dataset. The treatment with 80 nominal power showed an enrichment of up-regulated genes related to extracellular matrix organization and down-regulated genes corresponding to membrane protein intracellular domain proteolysis. The latter were identified using ToppGene Suite because IPA did not evidence relevant enrichment lists (S1 Fig).

### Assessment of gene expression after 40 QMR stimulation in quantitative real-time PCR

To confirm the gene expression modulation revealed by cDNA microarray analysis, quantitative real-time PCR was carried out in MSC cultures treated at 40 nominal power for one QMR cycle. As shown in Table 1, genes were involved in pathways related to cellular and tissue development, like ECM remodelling, angiogenesis, cellular migration and regulation of actin filaments. Differentially expressed genes obtained by 80 QMR treatment were not further investigated, due to the lower fold change and significance compared to 40 setting.

**Table 1:** Selected genes for testing DNA microarray outcomes in quantitative real-time PCR.

Gene name	Systematic name	P-value	Fold change	Protein name	Function
**MMP1**	NM_002421	0,00007	1,6	Interstitial collagenase	Cleaves collagens of types I, II, and III
**PLAT**	NM_000930	0,003	1,4	Tissue-type plasminogen activator	Role in tissue remodeling
**SLIT2**	NM_004787	0,003	1,3	Slit homolog 2 protein	Molecular guidance in cellular migration
**ARHGAP22**	NM_021226	0,004	1,3	Rho GTPase-activating protein 22	Regulates endothelial cell capillary tube formation
**A2M**	NM_000014	0,00005	-2,1	Alpha-2-macroglobulin	Inhibitor of proteinases
**CORO1B**	NM_020441	0,001	-1,4	Coronin-1B	Regulates leading edge dynamics and cell motility
**SHC1**	NM_183001	0,002	-1,5	SHC-transforming protein 1	Signaling adapter
**FN1**	NM_054034	0,005	-1,4	Fibronectin	Involved in cell adhesion and motility

The individuated genes took part to biological processes where MSC could have a role in the regenerative support after QMR stimulation.

**Fig 7:**
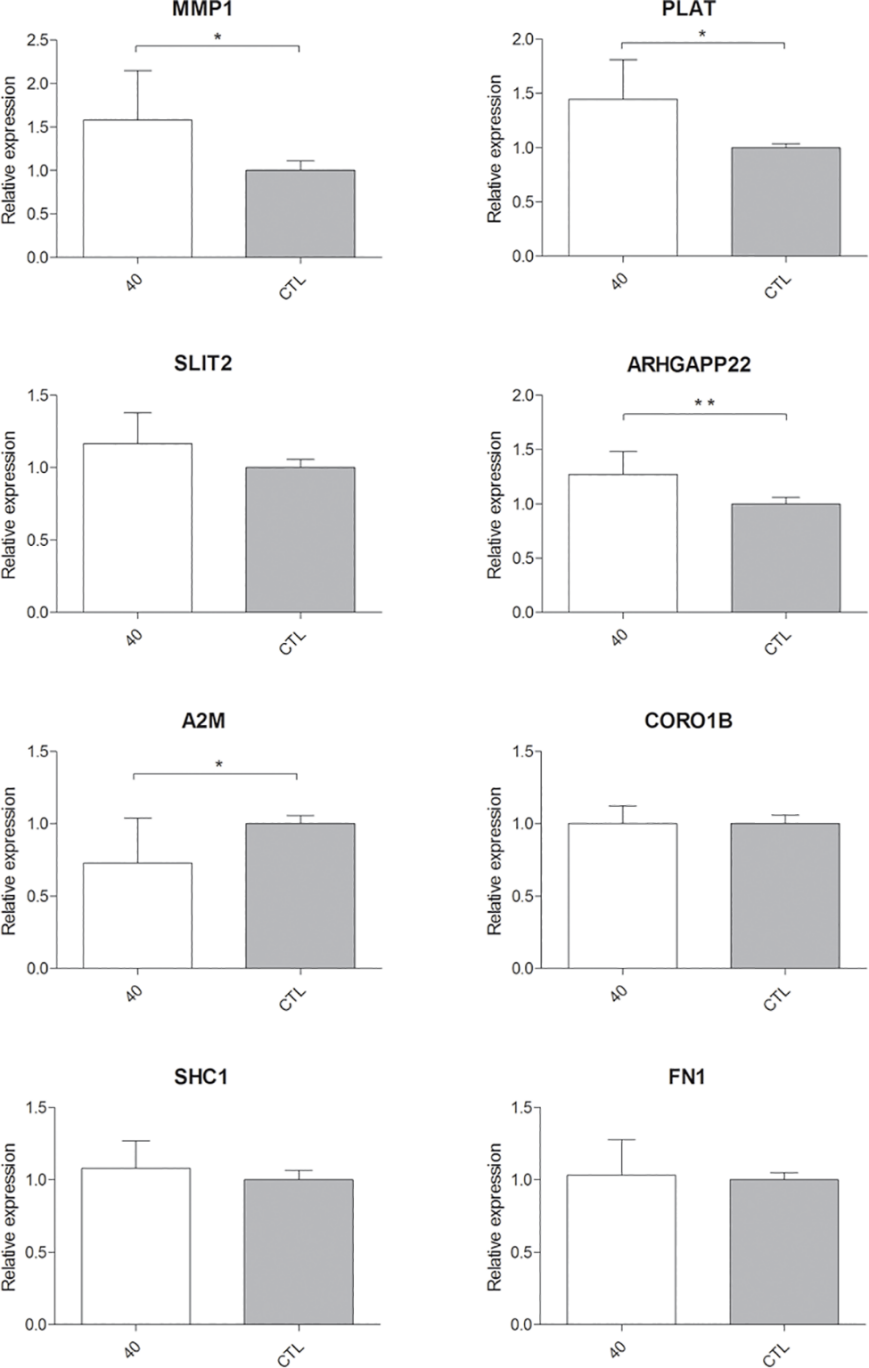
Relative gene expressions using quantitative real-time PCR. Expression of 8 genes selected by cDNA microarray was illustrated after n = 6 independent experiments using TBP as representative reference gene; mean ± SD; * p<0.05; ** p<0.01.

Our results from six independent experiments, revealed significant increased expression of MMP1, PLAT and ARHGAP22, while A2M gene showed significant decreased expression compared to the controls. By contrast SLIT2, CORO1B, SHC1 and FN1 were not found modulated by QMR treatment, partially confirming cDNA microarray data (Fig 7).

## Discussion

We analysed the effects of QMR treatment on MSC *in vitro* at cellular and molecular level.

At the cellular level, we have observed that the treatment with QMR maintained unchanged cell morphology and viability, cell phenotype at least based on cell markers analysis and cell proliferation.

It was evidenced that EMF and electric fields had the capacity to modify cell physiology and signalling pathways altering ion channels, transport protein activation and intracellular ionic concentration [1, 32]. In particular, some authors suggested that EMF affect early stages of differentiation and reduce the time of differentiation [33, 34]. Moreover, Teven and colleagues [35] demonstrated that high-frequency pulsed EMF stimulation augmented osteogenic differentiation. We observed that the ability of MSC exposed to QMR to generate mesodermal tissues *in vitro* was unaltered by the treatment.

To investigate a possible effect of QMR at the transcriptional level, we performed gene expression analysis. As expected for donor-derived cells, cDNA microarray analysis revealed high variability between the different MSC batches. This observation explained the low number of highly significant DEGs between different QMR conditions and controls. DEG analysis also revealed that MSC exposed at 40 QMR setting underwent to more transcriptional changes, suggesting that the treatment at this nominal power is more effective than 80 QMR. In both cases, the relatively low amplitude of the changes confirms the phenotypic observations. The reason because in our experimental setting the 40 stimulation was more effective than 80 remain unclear. In literature there are open questions regarding the mechanism of action of EMF [36]. Since a possible mechanism of action could be related to structural vibrations of electrically polar molecules or larger structures, it is likely that a molecule and a biological system could be more responsive to a particular intensity of stimulation in function of its polarity but further studies are necessary to clarify the issue. The gene set enrichment analysis of DEG, performed to understand which main biological processes were involved, revealed that QMR stimulation on MSC cultures affected a lot of different biofunctions. In fact, we found transcriptionally modulated genes related to development processes, regulation of phosphorylation, regulation of cellular pathways including metabolism, kinase activity and cellular organization. The most represented enrichment lists among up-regulated genes were related to cardiovascular system development as also observed by Serena et al [8] with the electrical stimulation of human embryonic stem cells. Sheikh and colleagues [37] showed that electric fields induced the regulation of endothelial antigenic response via MAPK/ERK pathway activation. In particular, our gene by gene analysis also revealed that 40 up- and down-regulated genes were involved in cellular and tissue development processes such as ECM remodelling, angiogenesis, cellular migration and regulation of actin filaments.

The most representative genes for each category were further validated by quantitative real-time PCR on MSC exposed to 40 nominal power after a single QMR cycle. Overall, 50% of them comprising ARHGAP22, MMP1, PLAT and A2M were found significantly modulated compared to controls. ARHGAP22 is a gene expressing a RhoGAP cytoplasmic protein involved in angiogenesis and in the negative regulation of rearrangement of actin filaments through the inhibition of Rac1 [38, 39]. This datum is interesting since some frequencies produced by QMR treatment are inside the endogenous range that affects actin and microtubule filaments [40].

Interestingly, differentially regulated MMP1, PLAT and A2M genes are involved in the ECM remodelling through the fibrinolytic system that is also implicated in embryogenesis, wound healing and angiogenesis [41].

PLAT is a serine protease that converts plasminogen into plasmin where the latter activates other proteases including MMP1 [41]. Neuss and collaborators [42] demonstrated that MSC were able to secrete enzymes involved into this biological pathway and our results showed its promotion by stimulated MSC. In particular, the positive regulation of the two enzymes PLAT (upstream protein) and MMP1 (downstream protein) was in agreement with the negative regulation of the inhibitor of proteases A2M.

Proteases participate in the regulation of angiogenesis through a modulation of an extremely complex process [43] whereas extracellular proteolysis is a requirement for new blood vessel formation. Therefore, matrix metalloproteinases as well as plasminogen activator-plasmin systems play an important role during angiogenesis [44, 45]. Their release allows the bioavailability of factors stored in ECM reservoir [46–48] and PLAT is able to activate PDGF-C [49]. Other studies demonstrated a direct induction of angiogenic factors using electric current [50–52].

In conclusion, our data suggests that in our model QMR-treated MSC maintained unaltered cell phenotype, viability, proliferation and the ability of MSC to differentiate into bone, cartilage and adipose tissue. cDNA microarray analysis may suggest an involvement of some genes after treatment in angiogenesis and in tissue regeneration probably through ECM remodelling. In the present study, donor-to-donor variability may have limited the robustness of microarray data to detect subtle modulation in gene expression profile. However, real-time PCR data validated changes detected in the highly regulated genes in QMR-treated MSC, relatively to the lower setting tested.

Further studies are necessary to confirm our findings both at the protein and at functional level on different cellular models.

## Supporting information

S1 Fig Best enrichment gene lists of up- and down- regulated DEG between 80 QMR setting and control using IPA software. Figure illustrated the comparative analysis of up-regulated (green bar) and down-regulated (red bar) functional gene enrichments using IPA software with significant enrichment (dotted line) for -log2 (B-H p-value) >1.3.
(TIF)

S2 Fig Relative median fluorescence intensity of MSC markers after QMR stimulation at different settings. A) First cycle of treatment; B) Second cycle of treatment. Bars represented the maximum, median and minimum values of 3 independent experiments. The y-axis was in log10 scale.
(TIF)

## References

[1] Liu Q, Song B. Electric field regulated signaling pathways. The international journal of biochemistry & cell biology. 2014 10;55:264–8. 25256684 10.1016/j.biocel.2014.09.014

[2] Taghian T, Narmoneva DA, Kogan AB. Modulation of cell function by electric field: a high-resolution analysis. J R Soc Interface. 2015 6;12(107). 10.1098/rsif.2015.0153 PMC4590499 25994294

[3] Costin GE, Birlea SA, Norris DA. Trends in wound repair: cellular and molecular basis of regenerative therapy using electromagnetic fields. Curr Mol Med. 2012 1;12(1):14–26. 22082478 10.2174/156652412798376143

[4] Fan W, Qian F, Ma Q, Zhang P, Chen T, Chen C, et al 50 Hz electromagnetic field exposure promotes proliferation and cytokine production of bone marrow mesenchymal stem cells. Int J Clin Exp Med. 2015;8(5):7394–404. 26221281 PMC4509226

[5] Zimmerman JW, Pennison MJ, Brezovich I, Yi N, Yang CT, Ramaker R, et al Cancer cell proliferation is inhibited by specific modulation frequencies. Br J Cancer. 2012 1;106(2):307–13. doi: 10.1038/bjc.2011.523 22134506 10.1038/bjc.2011.523 PMC3261663

[6] Stacey M, Fox P, Buescher S, Kolb J. Nanosecond pulsed electric field induced cytoskeleton, nuclear membrane and telomere damage adversely impact cell survival. Bioelectrochemistry. 2011 10;82(2):131–4. doi: 10.1016/j.bioelechem.2011.06.002 21719360 10.1016/j.bioelechem.2011.06.002

[7] Yoon YJ, Li G, Kim GC, Lee HJ, Song K. Effects of 60-Hz time-varying electric fields on DNA damage and cell viability support negligible genotoxicity of the electric fields. JEES. 2015;15(3):134–41.

[8] Serena E, Figallo E, Tandon N, Cannizzaro C, Gerecht S, Elvassore N, et al Electrical stimulation of human embryonic stem cells: cardiac differentiation and the generation of reactive oxygen species. Exp Cell Res. 2009 12;315(20):3611–9. doi: 10.1016/j.yexcr.2009.08.015 19720058 10.1016/j.yexcr.2009.08.015 PMC2787733

[9] Rouabhia M, Park H, Meng S, Derbali H, Zhang Z. Electrical stimulation promotes wound healing by enhancing dermal fibroblast activity and promoting myofibroblast transdifferentiation. PLoS One. 2013;8(8):e71660 doi: 10.1371/journal.pone.0071660 23990967 10.1371/journal.pone.0071660 PMC3747189

[10] Maioli M, Rinaldi S, Santaniello S, Castagna A, Pigliaru G, Gualini S, et al Radiofrequency energy loop primes cardiac, neuronal, and skeletal muscle differentiation in mouse embryonic stem cells: a new tool for improving tissue regeneration. Cell Transplant. 2012;21(6):1225–33. doi: 10.3727/096368911X600966 21975035 10.3727/096368911X600966

[11] Zhao Z, Watt C, Karystinou A, Roelofs AJ, McCaig CD, Gibson IR, et al Directed migration of human bone marrow mesenchymal stem cells in a physiological direct current electric field. Eur Cell Mater. 2011 11;22:344–58. 22125259 10.22203/ecm.v022a26

[12] Tandon N, Goh B, Marsano A, Chao PH, Montouri-Sorrentino C, Gimble J, et al Alignment and elongation of human adipose-derived stem cells in response to direct-current electrical stimulation. Conf Proc IEEE Eng Med Biol Soc. 2009;2009:6517–21. doi: 10.1109/IEMBS.2009.5333142 19964171 10.1109/IEMBS.2009.5333142 PMC2791914

[13] Zhang J, Ren R, Luo X, Fan P, Liu X, Liang S, et al A small physiological electric field mediated responses of extravillous trophoblasts derived from HTR8/SVneo cells: involvement of activation of focal adhesion kinase signaling. PLoS One. 2014;9(3):e92252 doi: 10.1371/journal.pone.0092252 24643246 10.1371/journal.pone.0092252 PMC3958492

[14] Gómez-Ochoa I, Gómez-Ochoa P, Gómez-Casal F, Cativiela E, Larrad-Mur L. Pulsed electromagnetic fields decrease proinflammatory cytokine secretion (IL-1β and TNF-α) on human fibroblast-like cell culture. Rheumatol Int. 2011 10;31(10):1283–9. doi: 10.1007/s00296-010-1488-0 20372910 10.1007/s00296-010-1488-0

[15] Vianale G, Reale M, Amerio P, Stefanachi M, Di Luzio S, Muraro R. Extremely low frequency electromagnetic field enhances human keratinocyte cell growth and decreases proinflammatory chemokine production. Br J Dermatol. 2008 6;158(6):1189–96. doi: 10.1111/j.1365-2133.2008.08540.x 18410412 10.1111/j.1365-2133.2008.08540.x

[16] Jennings J, Chen D, Feldman D. Transcriptional response of dermal fibroblasts in direct current electric fields. Bioelectromagnetics. 2008 7;29(5):394–405. doi: 10.1002/bem.20408 18302142 10.1002/bem.20408

[17] Lee HC, Hong MN, Jung SH, Kim BC, Suh YJ, Ko YG, et al Effect of extremely low frequency magnetic fields on cell proliferation and gene expression. Bioelectromagnetics. 2015 10;36(7):506–16. doi: 10.1002/bem.21932 26239017 10.1002/bem.21932

[18] Messerli MA, Graham DM. Extracellular electrical fields direct wound healing and regeneration. Biol Bull. 2011 8;221(1):79–92. doi: 10.1086/BBLv221n1p79 21876112 10.1086/BBLv221n1p79

[19] D'Eredità R, Bozzola L. Molecular resonance vs. coblation tonsillectomy in children. Laryngoscope. 2009 10;119(10):1897–901. doi: 10.1002/lary.20210 19598217 10.1002/lary.20210

[20] Chang H, Hah JH. Comparison of post-tonsillectomy pain with two different types of bipolar forceps: low temperature quantum molecular resonance device versus high temperature conventional electrocautery. Acta Otolaryngol. 2012 6;132 Suppl 1:S130–3. 22384925 10.3109/00016489.2012.659752

[21] Schiavon M, Calabrese F, Nicotra S, Marulli G, Pozzato G, Giacometti C, et al Favorable tissue effects of quantum molecular resonance device (Vesalius) compared with standard electrocautery. A novel paradigm in lung surgery. Eur Surg Res. 2007;39(4):222–8. doi: 10.1159/000101745 17438358 10.1159/000101745

[22] Dal Maschio M, Canato M, Pigozzo FM, Cipullo A, Pozzato G, Reggiani C. Biophysical effects of high frequency electrical field (4–64 MHz) on muscle fibers in culture. Basic Applied Miology. 2009;19(1):49–56.

[23] Lopresti M, Tomba A, Caserta A, Di Dimenica F. Effectiveness of molecular quantum resonance in reducing edema after total knee arthroplasty: a clinical trial. Arch Ortop Reumatol. 2011;122(1):34–5.

[24] Murphy MB, Moncivais K, Caplan AI. Mesenchymal stem cells: environmentally responsive therapeutics for regenerative medicine. Exp Mol Med. 2013 11;45:e54 doi: 10.1038/emm.2013.94 24232253 10.1038/emm.2013.94 PMC3849579

[25] Rubio-Azpeitia E, Andia I. Partnership between platelet-rich plasma and mesenchymal stem cells: in vitro experience. Muscles Ligaments Tendons J. 2014 1;4(1):52–62. 24932448 PMC4049651

[26] Dominici M, Le Blanc K, Mueller I, Slaper-Cortenbach I, Marini F, Krause D, et al Minimal criteria for defining multipotent mesenchymal stromal cells. The International Society for Cellular Therapy position statement. Cytotherapy. 2006;8(4):315–7. doi: 10.1080/14653240600855905 16923606 10.1080/14653240600855905

[27] Aggarwal S, Pittenger MF. Human mesenchymal stem cells modulate allogeneic immune cell responses. Blood. 2005 2 15;105(4):1815–22. doi: 10.1182/blood-2004-04-1559 15494428 10.1182/blood-2004-04-1559

[28] Uccelli A, Moretta L, Pistoia V. Immunoregulatory function of mesenchymal stem cells. Eur J Immunol. 2006 10;36(10):2566–73. doi: 10.1002/eji.200636416 17013987 10.1002/eji.200636416

[29] Chen Y, Xiang L-X, Shao J-Z, Pan R-L, Wang Y-X, Dong X-J, et al Recruitment of endogenous bone marrow mesenchymal stem cells towards injured liver. Journal of Cellular and Molecular Medicine. 2010;14(6b):1494–508. doi: 10.1111/j.1582-4934.2009.00912.x 19780871 10.1111/j.1582-4934.2009.00912.x PMC3829016

[30] Ritchie ME, Phipson B, Wu D, Hu Y, Law CW, Shi W, et al limma powers differential expression analyses for RNA-sequencing and microarray studies. Nucleic Acids Res. 2015 4;43(7):e47 doi: 10.1093/nar/gkv007 25605792 10.1093/nar/gkv007 PMC4402510

[31] Livak KJ, Schmittgen TD. Analysis of relative gene expression data using real-time quantitative PCR and the 2(-Delta Delta C(T)) Method. Methods. 2001 12;25(4):402–8. doi: 10.1006/meth.2001.1262 11846609 10.1006/meth.2001.1262

[32] Sundelacruz S, Levin M, Kaplan DL. Membrane potential controls adipogenic and osteogenic differentiation of mesenchymal stem cells. PLoS One. 2008;3(11):e3737 doi: 10.1371/journal.pone.0003737 19011685 10.1371/journal.pone.0003737 PMC2581599

[33] Sun LY, Hsieh DK, Lin PC, Chiu HT, Chiou TW. Pulsed electromagnetic fields accelerate proliferation and osteogenic gene expression in human bone marrow mesenchymal stem cells during osteogenic differentiation. Bioelectromagnetics. 2010 4;31(3):209–19. doi: 10.1002/bem.20550 19866474 10.1002/bem.20550

[34] Esposito M, Lucariello A, Riccio I, Riccio V, Esposito V, Riccardi G. Differentiation of human osteoprogenitor cells increases after treatment with pulsed electromagnetic fields. In Vivo. 2012 2012 Mar-Apr;26(2):299–304. 22351673

[35] Teven CM, Greives M, Natale RB, Su Y, Luo Q, He BC, et al Differentiation of osteoprogenitor cells is induced by high-frequency pulsed electromagnetic fields. J Craniofac Surg. 2012 3;23(2):586–93. doi: 10.1097/SCS.0b013e31824cd6de 22446422 10.1097/SCS.0b013e31824cd6de

[36] Kučera O, Cifra M. Radiofrequency and microwave interactions between biomolecular systems. Journal of Biological Physics. 2016;42(1):1–8. doi: 10.1007/s10867-015-9392-1 26174548 10.1007/s10867-015-9392-1 PMC4713408

[37] Sheikh AQ, Taghian T, Hemingway B, Cho H, Kogan AB, Narmoneva DA. Regulation of endothelial MAPK/ERK signalling and capillary morphogenesis by low-amplitude electric field. J R Soc Interface. 2013 Jan;10(78):20120548 doi: 10.1098/rsif.2012.0548 22993248 10.1098/rsif.2012.0548 PMC3565781

[38] Marinković G, Heemskerk N, van Buul JD, de Waard V. The Ins and Outs of Small GTPase Rac1 in the Vasculature. J Pharmacol Exp Ther. 2015 8;354(2):91–102. doi: 10.1124/jpet.115.223610 26036474 10.1124/jpet.115.223610

[39] Mori M, Saito K, Ohta Y. ARHGAP22 localizes at endosomes and regulates actin cytoskeleton. PLoS One. 2014;9(6):e100271 doi: 10.1371/journal.pone.0100271 24933155 10.1371/journal.pone.0100271 PMC4059726

[40] Cifra M, Fields JZ, Farhadi A. Electromagnetic cellular interactions. Prog Biophys Mol Biol. 2011 5;105(3):223–46. doi: 10.1016/j.pbiomolbio.2010.07.003 20674588 10.1016/j.pbiomolbio.2010.07.003

[41] Heissig B, Dhahri D, Eiamboonsert S, Salama Y, Shimazu H, Munakata S, et al Role of mesenchymal stem cell-derived fibrinolytic factor in tissue regeneration and cancer progression. Cell Mol Life Sci. 2015 12;72(24):4759–70. doi: 10.1007/s00018-015-2035-7 26350342 10.1007/s00018-015-2035-7 PMC11113371

[42] Neuss S, Schneider RK, Tietze L, Knüchel R, Jahnen-Dechent W. Secretion of fibrinolytic enzymes facilitates human mesenchymal stem cell invasion into fibrin clots. Cells Tissues Organs. 2010;191(1):36–46. doi: 10.1159/000215579 19390164 10.1159/000215579

[43] Roy R, Zhang B, Moses MA. Making the cut: protease-mediated regulation of angiogenesis. Exp Cell Res. 2006 3;312(5):608–22. doi: 10.1016/j.yexcr.2005.11.022 16442099 10.1016/j.yexcr.2005.11.022

[44] Pepper MS. Role of the matrix metalloproteinase and plasminogen activator-plasmin systems in angiogenesis. Arterioscler Thromb Vasc Biol. 2001 7;21(7):1104–17. 11451738 10.1161/hq0701.093685

[45] Nam HS, Kwon I, Lee BH, Kim H, Kim J, An S, et al Effects of Mesenchymal Stem Cell Treatment on the Expression of Matrix Metalloproteinases and Angiogenesis during Ischemic Stroke Recovery. PLoS One. 2015;10(12):e0144218 doi: 10.1371/journal.pone.0144218 26637168 10.1371/journal.pone.0144218 PMC4670145

[46] Park JE, Keller GA, Ferrara N. The vascular endothelial growth factor (VEGF) isoforms: differential deposition into the subepithelial extracellular matrix and bioactivity of extracellular matrix-bound VEGF. Mol Biol Cell. 1993 12;4(12):1317–26. 8167412 10.1091/mbc.4.12.1317 PMC275767

[47] Whitelock JM, Murdoch AD, Iozzo RV, Underwood PA. The degradation of human endothelial cell-derived perlecan and release of bound basic fibroblast growth factor by stromelysin, collagenase, plasmin, and heparanases. J Biol Chem. 1996 4;271(17):10079–86. 8626565 10.1074/jbc.271.17.10079

[48] Bonnans C, Chou J, Werb Z. Remodelling the extracellular matrix in development and disease. Nat Rev Mol Cell Biol. 2014 12;15(12):786–801. doi: 10.1038/nrm3904 25415508 10.1038/nrm3904 PMC4316204

[49] Fredriksson L, Li H, Fieber C, Li X, Eriksson U. Tissue plasminogen activator is a potent activator of PDGF-CC. EMBO J. 2004 10;23(19):3793–802. doi: 10.1038/sj.emboj.7600397 15372073 10.1038/sj.emboj.7600397 PMC522796

[50] Kim IS, Song JK, Zhang YL, Lee TH, Cho TH, Song YM, et al Biphasic electric current stimulates proliferation and induces VEGF production in osteoblasts. Biochim Biophys Acta. 2006 9;1763(9):907–16. doi: 10.1016/j.bbamcr.2006.06.007 16930744 10.1016/j.bbamcr.2006.06.007

[51] Bai H, Forrester JV, Zhao M. DC electric stimulation upregulates angiogenic factors in endothelial cells through activation of VEGF receptors. Cytokine. 2011 7;55(1):110–5. doi: 10.1016/j.cyto.2011.03.003 21524919 10.1016/j.cyto.2011.03.003 PMC4437767

[52] Asadi MR, Torkaman G, Hedayati M, Mofid M. Role of sensory and motor intensity of electrical stimulation on fibroblastic growth factor-2 expression, inflammation, vascularization, and mechanical strength of full-thickness wounds. J Rehabil Res Dev. 2013;50(4):489–98. 23934870 10.1682/jrrd.2012.04.0074

