# Supplementary figures and images for: In-vitro analysis of Quantum Molecular Resonance effects on human mesenchymal stromal cells
Source: PLoS One. 2018 Jan 2;13(1):e0190082. doi: 10.1371/journal.pone.0190082 (PMC5749755; doi:10.1371/journal.pone.0190082)

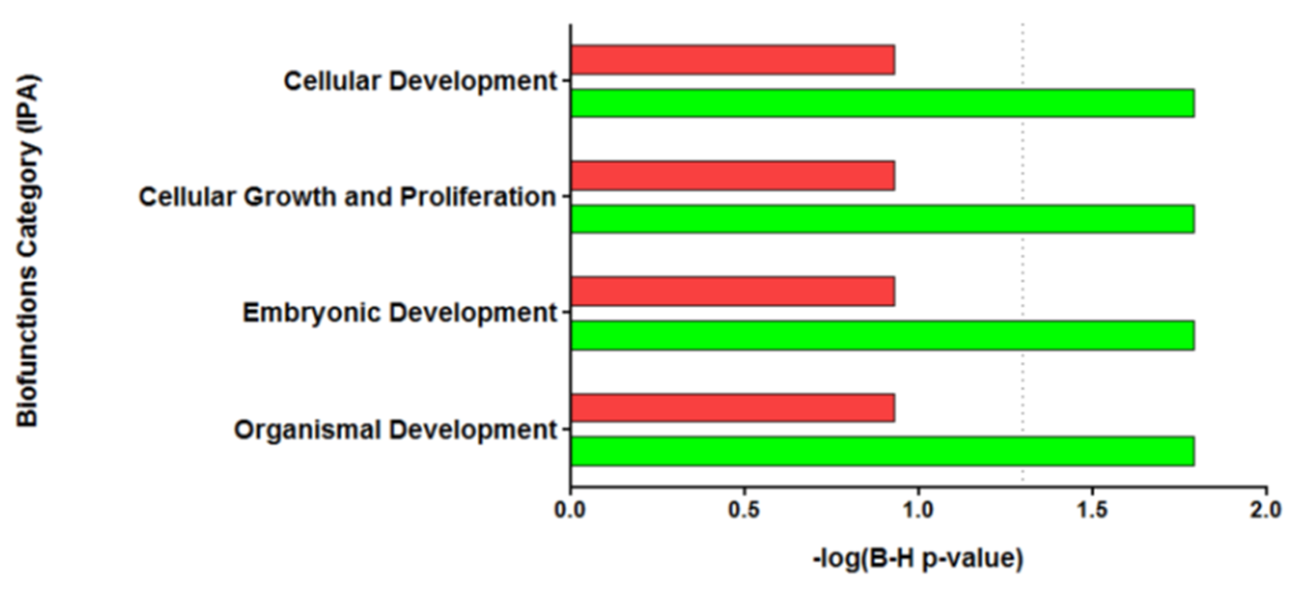

Supplement: S1 Fig — Figure illustrated the comparative analysis of up-regulated (green bar) and down-regulated (red bar) functional gene enrichments using IPA software with significant enrichment (dotted line) for -log2 (B-H p-value) >1.3. (TIF) [file pone.0190082.s001.tif]

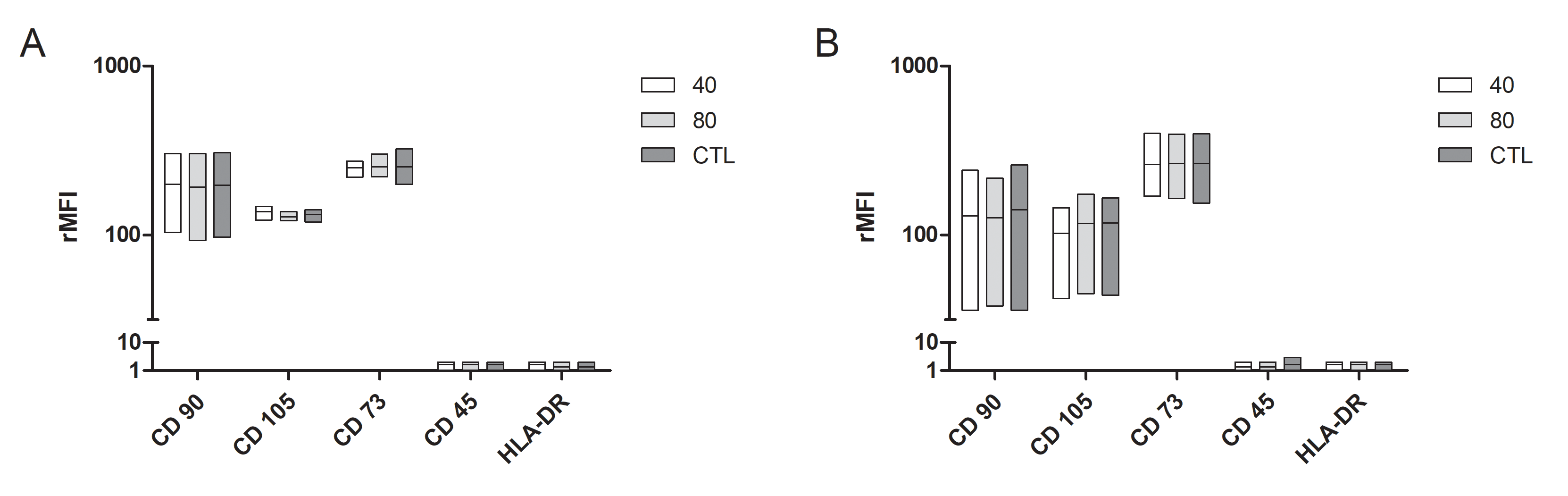

Supplement: S2 Fig — A) First cycle of treatment; B) Second cycle of treatment. Bars represented the maximum, median and minimum values of 3 independent experiments. The y-axis was in log10 scale. (TIF) [file pone.0190082.s002.tif]
